# Seeing Through Feeling: Dynamic Interplay Between Emotion and Visual Perception

**DOI:** 10.3390/brainsci16070696

**Published:** 2026-06-30

**Authors:** Nika Vukosav, Krista Zuber, Sara Tomas, Vanja Kopilaš

**Affiliations:** Faculty of Croatian Studies, University of Zagreb, 10000 Zagreb, Croatia; nvukosav@fhs.hr (N.V.); kzuber@fhs.hr (K.Z.); stomas@fhs.hr (S.T.)

**Keywords:** visual perception, affective neuroscience, amygdala, predictive coding, active inference, oculomics, psychopathology

## Abstract

For decades, visual perception was treated as a linear, feature-extracting mechanism driven almost exclusively by bottom-up sensory inputs. Emerging insights from affective neuroscience and cognitive psychology have systematically dismantled this view, revealing that vision operates within a continuous, bidirectional dialog with emotional systems. This review synthesizes the multi-layered neurobiological architectures underpinning this relationship. The pathways through which top–down emotional states recalibrate sensory processing are analyzed. Mechanisms including amygdalocortical feedback, frontoparietal attentional networks, and insular interoceptive monitoring are examined. These systems prioritize survival-driven motivational salience over objective accuracy. In the opposite direction, the text charts how ambient environmental features, such as lighting dynamics, spatial geometry, and structural ambiguity, immediately register along rapid subcortical and detailed cortical streams to instantiate emotional states. By situating these reciprocal dynamics within predictive coding and active inference frameworks, this paper illustrates how affective states function as precision weights that dynamically adjust internal perceptual priors. Finally, the clinical utility of these interconnected systems is evaluated, demonstrating how subtle visual aberrations like disrupted contrast suppression serve as diagnostic signatures for mood disorders, while structural retinal decay offers an accessible window into neurodegenerative pathology. Ultimately, the evidence indicates that conscious vision is fundamentally an affective construction, carrying transformative implications for early biomathematical and ocular screening in psychopathology.

## 1. Introduction

Vision is often regarded as a window into the external world, yet what is seen is never entirely independent of what is felt. As the dominant human sensory modality, vision unfolds in continuous dialog with affective systems that imbue perception with meaning, relevance, and urgency. Rather than passively registering incoming stimuli, the visual system operates as a selective and interpretive process, shaped by emotional states that guide attention toward what matters most, such as signals of threat, opportunity, or social significance. A fleeting glance can thus be weighted with fear, desire, or aversion, illustrating that perception is not merely about detecting the world, but about evaluating it. Equally, the relationship runs in the opposite direction: visual experiences have the capacity to evoke, amplify, and transform emotional states, often with remarkable immediacy. This bidirectional interplay between seeing and feeling is fundamental to adaptive functioning, enabling organisms to navigate complex environments by rapidly integrating sensory input with affective value. Without such coupling, perception would risk becoming indifferent to context, stripped of the prioritization necessary for survival and meaningful interaction. Contemporary research in psychology and neuroscience increasingly supports the view that visual and emotional processes are deeply intertwined. These processes rely on overlapping neural systems that interact across multiple levels of analysis [1,2,3,4,5,6]. Understanding this reciprocal relationship is therefore essential for any comprehensive account of perception. It suggests that vision is not a detached reflection of reality, but an active construction shaped by the organism’s internal landscape as much as by the external world. Building on this perspective, this paper examines how emotional and visual systems interact to produce a perceptual experience that is simultaneously sensory and affective, immediate yet interpretive. This narrative review synthesizes evidence regarding the bidirectional interaction between visual perception and affective neurobiology. A structured literature search was conducted across the PubMed, PsycINFO, and Web of Science databases using keywords encompassing affective modulation, predictive processing, and clinical oculomics. Given the narrative nature of this review, article selection was determined by thematic relevance to the intersection of computational perception and psychopathology, without the application of formal quality assessment or predefined inclusion criteria.

## 2. The Affective Modulation of Visual Attention and Processing

Emotional states exert a measurable influence on visual perception, modulating both the selection and interpretation of sensory input. Converging evidence from behavioral and neurophysiological studies indicates that affective significance can bias attentional allocation toward emotionally salient stimuli, often at the expense of neutral information [7]. Moreover, early stages of visual processing appear to be shaped by feedback from limbic and prefrontal regions, suggesting that perception is not a purely bottom–up process but is dynamically integrated with emotional evaluation [8,9]. Such interactions enable rapid prioritization of biologically relevant cues, thereby enhancing adaptive responses in complex environments.

Emotions exert a profound and systematic influence on visual perception through multilayered neurobiological mechanisms that integrate rapid subcortical appraisal with modulatory cortical control, resulting in a perceptual system that is inherently selective rather than veridical [10,11]. Central to this process is the amygdala, which functions as a hub for detecting emotionally salient stimuli and orchestrating their prioritized processing [12,13,14,15]. The system receives input via a fast, subcortical pathway relayed through the superior colliculus and pulvinar, as well as a slower, cortically mediated route, which allows for early, low resolution threat detection alongside more refined perceptual analysis. Crucially, the amygdala exerts modulatory feedback on multiple levels of the visual cortex, from primary visual areas (V1) to higher-order extrastriate regions. This feedback amplifies the representation of emotionally relevant stimuli, biasing perceptual competition [12]. The pulvinar coordinates these processes by regulating thalamocortical communication and synchronizing oscillatory activity between visual regions and frontoparietal attention networks, which facilitates selective attention toward salient inputs [12,16]. The superior colliculus contributes to this system by mediating rapid orienting responses and saccadic eye movements, ensuring that potentially significant stimuli are brought into the focus of high-acuity vision [12,17].

At the cortical level, top–down influences are primarily mediated by the prefrontal cortex (PFC), particularly its dorsolateral and ventromedial subdivisions, which implement executive control over attention and perception [18]. These regions dynamically bias sensory processing in accordance with current goals, expectations, and emotional states, while also exerting regulatory control over limbic structures such as the amygdala. The insula further integrates interoceptive information into perceptual experience, contributing to the subjective intensity and relevance of visual stimuli. In parallel, category-selective regions such as the fusiform face area (FFA) demonstrate heightened sensitivity to emotionally expressive faces, illustrating how affective significance can modulate specialized perceptual systems [18,19,20]. These distributed processes are embedded within larger-scale salience and attentional networks, which continuously evaluate incoming sensory information and allocate processing resources based on its motivational and emotional importance.

Converging psychological evidence supports this neurobiological framework. Mood-congruent perceptual biases indicate that individuals are more likely to attend to and interpret stimuli in ways that are consistent with their current affective state, reflecting top–down modulation of perceptual processing [21]. Similarly, visual search paradigms robustly demonstrate the “threat superiority effect,” wherein threatening stimuli are detected more rapidly and efficiently than neutral counterparts, even under conditions of limited attentional resources [22]. This phenomenon is widely interpreted as an evolutionarily adaptive mechanism, supported by neural circuits optimized for rapid threat detection [22,23]. Importantly, such findings highlight that the visual system prioritizes salience over objective accuracy because it is functionally more advantageous to generate false positives than to fail to detect a genuine threat [24,25]. This asymmetry reflects a broader principle of perceptual organization in which relevance, rather than fidelity, governs processing priorities.

Within this framework, perceptual biases are not merely distortions or errors but constitute adaptive responses to environmental demands. Emotional states systematically recalibrate perceptual sensitivity. Although vision–emotion interactions have been investigated across a range of psychiatric conditions, this review focuses primarily on anxiety and major depressive disorder because they are among the most prevalent mental disorders worldwide and have been the most extensively studied in relation to visual perception. These disorders therefore provide the strongest empirical basis for examining how emotional states influence visual processing and its potential clinical implications. Anxiety is associated with heightened vigilance and increased responsivity within amygdala-centered salience networks, leading to an enhanced detection of threat-related cues, whereas depression is linked to attenuated perceptual responsiveness and reduced neural gain, contributing to diminished engagement with environmental stimuli [12,14,26,27]. These alterations underscore the dynamic and context dependent nature of perception, which emerges from continuous interactions between bottom-up sensory input and top–down emotional and cognitive influences. Taken together, contemporary evidence demonstrates that vision is not a passive, stimulus-driven process but an active, inferential system shaped by the organism’s affective priorities, in which emotional significance fundamentally determines what is seen, how it is processed, and ultimately, how it is experienced. The integration of subcortical appraisal and cortical control mechanisms reveals that visual perception functions as an active, inferential system rather than a passive reflection of reality. By prioritizing affective salience over objective fidelity, the visual system demonstrates how emotional states fundamentally calibrate sensory sensitivity to meet adaptive demands, confirming that perception is an inherently selective process driven by the organism’s motivational priorities.

## 3. Emotional Amplification

Emotional amplification refers to the idea that emotions can intensify the way visual information is noticed, experienced, and remembered [28,29]. Emotional stimuli often receive priority in attention, especially when they are relevant to the current situation. Emotionally arousing stimuli capture and sustain attention during task-relevant processing, potentially enhancing performance through increased speed or precision [30]. However, when emotional stimuli are irrelevant, they can distract attention and impair performance. This shows that emotional amplification is selective: emotions do not simply improve perception in general, but prioritize information that seems important or meaningful. Emotions can also change the subjective appearance of visual stimuli. Emotion and anxiety amplify the influence of exogenous attention on perceived contrast [31]. In other words, emotional cues, such as fearful faces, can make later visual stimuli appear more contrastive when they appear in the same location. The effects of emotional amplification can continue into memory. Emotional visual scenes are often remembered differently from neutral scenes because emotion directs attention toward the most important parts of the image. Emotional content enhances memory for central affective elements while simultaneously impairing the retention of neutral background information [32]. This means that emotion does not strengthen all parts of a visual scene equally, but selectively emphasizes what appears most significant. Furthermore, emotional states modulate temporal memory, effectively shaping the subjective organization of events in time [33]. Continuous negative events may make remembered time feel compressed, while sudden shifts from neutral to negative events may make time feel expanded.

Depression is a good example of how emotions and mood can affect not only the way a person thinks, but also the way they visually perceive the world. People with depression often show a negative perceptual bias, meaning that they tend to interpret neutral or ambiguous stimuli more negatively than people without depression. This is especially visible in face perception. Individuals with major depressive disorder exhibit a bias toward attributing negative valence to neutral facial expressions [34], frequently interpreting them as sad rather than the positive interpretations typically favored by healthy control subjects [35]. This suggests that depression can change the emotional meaning of what a person sees, even when the stimulus itself is not clearly negative. This bias is not limited to emotion recognition, but also appears in the esthetic evaluation of faces. Individuals with depression demonstrate a negative evaluation bias, characterized by a higher frequency of rating faces as unattractive and a lower frequency of rating them as attractive compared to healthy controls [36]. Interestingly, this effect appeared for faces, but not for landscapes, suggesting that depression may especially affect the processing of socially important visual stimuli. In addition, people with depression tend to maintain their attention on sad faces for longer, which may reflect increased processing of negative content and difficulties disengaging attention from negative information [37].

From a neuropsychological perspective, these findings suggest that depression may affect visual perception not only at the level of conscious interpretation, but also during earlier stages of sensory processing. Research shows that people with depression may have a weaker neural response to visual stimuli, reflected in a reduced vertex positive potential (VPP) [38]. This suggests altered early processing of visual information and may indicate that attention is partly shifted away from external stimuli and toward internal processes, such as rumination [38]. Studies of contrast processing also show that depression can be associated with reduced contrast suppression, meaning that basic visual mechanisms involved in detecting contrast may function differently during depressive episodes [39,40]. These findings help explain why visual experience in depression may feel less vivid or more negatively colored. This does not mean that people with depression literally see the world in gray, but that some aspects of visual processing, such as contrast, inhibition, and motion perception, can be measurably altered. Research found that acute major depressive disorder may be associated with reduced gamma-aminobutyric acid (GABA) levels in higher-order occipital visual areas, which corresponds to impaired visual motion perception [41]. Since GABA is important for neural inhibition, these findings suggest that depression may disturb the balance between excitation and inhibition in the visual cortex. More recent research also links major depressive disorder to abnormal activation patterns in the middle temporal complex (MT+ visual area), which is involved in motion perception, further supporting the idea that depression can affect basic visual mechanisms, not only emotional judgment [42]. Together, this evidence shows that depression can shape visual perception at multiple levels: from early sensory processing, through attention, to interpretation and emotional meaning. Therefore, depression does not only influence how people feel about what they see, but may also change how visual information is processed, and given significance before it becomes part of conscious experience. This perspective is further supported by clinical research indicating that objective sensory limitations and somatic ocular distress significantly covary with heightened indicators of depression, anxiety, and perceived psychological stress, suggesting that visual impairment acts as both a biomarker and an exacerbating factor for poor mental health outcomes [43]. While these findings show how emotional states can alter visual processing and amplify the significance of what is seen, they also point to the opposite side of this relationship, in which visual input itself can shape emotional experience.

## 4. Visual Modulation of Emotional Experience

Beyond understanding how emotions shape our perception, it is equally important to examine the inverse impact of visual input on emotional experience. Visual input is not simply registered as a neutral representation of the external world, but is continuously processed and interpreted through neural systems that give it affective significance. Examining this relationship is especially important because it helps explain how visual information becomes linked to emotional evaluation, memory, and behavioral responses. This perspective also provides a broader framework for examining the biological basis of vision-emotion interactions and related phenomena, including the effects of color on emotion, the role of vision in emotional memory, and the contribution of visual processing to affective experience.

Vision is crucial for emotions because it enables humans to detect emotionally relevant features of the environment, such as threat, facial expressions, eye contact, and other socially significant cues, thereby rapidly directing attention and shaping emotional evaluation and response [44,45]. Through this process, visual perception helps determine what is experienced as important, pleasant, threatening, or emotionally significant, and thus plays a direct role in the emergence and direction of emotional experience [46].

Visual information begins in the eye, where the retina transforms light into neural signals. These signals are then sent through the thalamus to the visual cortex, where basic features such as shape, color, contrast, edges, and motion are processed. From there, visual information is connected with wider emotional and attentional networks, including limbic structures involved in emotional evaluation, memory, motivation, and bodily responses [46,47]. This connection between vision and emotion allows visual stimuli to be processed through parallel pathways. The slower cortical pathway involves detailed processing in the visual cortex and supports conscious interpretation of what we see. At the same time, some visual information may also reach emotional structures through a faster subcortical route involving the superior colliculus, pulvinar, and amygdala. This route is less detailed, but it allows the brain to make a quick and rough evaluation of possible danger before full conscious analysis takes place [15,47]. Although the subcortical pathway is frequently cited as a mechanism for rapid affective detection, its functional autonomy is debated, with alternative frameworks highlighting that cortical feedback is essential for detailed stimulus categorization [48].

The amygdala is especially important because it helps detect emotionally relevant visual cues, particularly signs of threat such as fearful or angry faces. These cues can quickly attract attention and prepare the body for action, even before a person fully understands what they are seeing. Research shows that fear-related signals are prioritized in visual and spatial processing, meaning that threatening visual cues are more likely to capture attention and guide behavior [49]. Body language can work in a similar way: tense posture, defensive movement, or panic-like motion can communicate emotional meaning and trigger automatic physiological responses, such as changes in pupil size [50].

Overall, these bottom-up emotional responses show that some emotional reactions are rapid, automatic, and partly unconscious. Visual cues, such as facial expressions and body language can activate limbic pathways, guide attention, and prepare the body for reaction before slower conscious interpretation is complete [15,49,50]. In this way, what we see can begin to shape how we feel almost immediately. The same principle extends beyond individual visual cues, as the overall atmosphere of a scene can also influence how visual experience acquires emotional meaning.

## 5. The Emotional Role of Visual Atmosphere

The visual environment affects emotions not only through what we recognize, but also through how a scene is visually shaped. The brain uses these cues very quickly to evaluate whether a situation feels pleasant, or potentially threatening, so an emotional reaction can appear before we consciously analyze the scene. In this sense, vision is not a passive transfer of an image, but a system that constantly links perceptual information with attention, arousal, and emotional meaning [51]. Color is a clear example of this because it can change the basic emotional tone of a space or scene. Warm colors, such as red, orange, and yellow, are often associated with higher activation, and warning, while cool colors, such as blue and green, are more often associated with calmness, and lower tension [52]. This does not mean that color directly produces an emotion, but rather that it gives the brain additional context for interpreting what we see. For example, red tones may strengthen the impression of anger or threat, while blue tones may guide the viewer toward a calmer interpretation of the scene [51].

From a neuroscientific perspective, color is not processed completely separately from emotional and bodily responses. Research on color in the built environment has shown that blue can influence autonomic activity, skin conductance, and frontal EEG patterns related to emotional processing, suggesting that color may affect both subjective experience and physiological arousal [53]. This physiological response is mediated by intrinsically photosensitive retinal ganglion cells (ipRGCs), a specialized class of retinal cells that differs from conventional photoreceptors such as rods and cones. These cells are particularly responsive to blue wavelengths of light and transmit signals directly to the suprachiasmatic nucleus (SCN) within the hypothalamus. Through this neural pathway, environmental light and color influence the autonomic nervous system, thereby contributing to the regulation of cortisol secretion, heart rate variability, and core body temperature [54,55]. In other words, when we say that a space feels calm or tense, this is not necessarily just a metaphor, but may reflect real changes in the body’s level of arousal. Similarly, research on individuals with red-green color blindness shows that emotional associations with colors do not depend only on the direct perception of color, but also on learned meanings and language [56].

Light is another important channel through which vision shapes emotions. Light intensity and color temperature can influence mood, alertness, and the speed of emotional evaluation of a scene. Research found that warmer lighting reduced negative response bias, meaning that participants were less likely to label ambiguous faces as fearful [57]. This suggests that lighting can influence not only general mood, but also the way emotionally unclear social information is interpreted. Stronger and clearer lighting usually increases the sense of control because a person can more easily recognize faces, obstacles, exits, and possible sources of danger. In contrast, poorly lit spaces create more uncertainty because the brain has to fill in missing information, and that uncertainty can increase discomfort or fear [58]. This spatial uncertainty triggers a state of hypervigilance. Reduced visual clarity is associated with increased cortical arousal through engagement of the ascending reticular activating system (RAS), and the locus coeruleus–norepinephrine system. Together, these mechanisms enhance vigilance, facilitate the detection of potentially significant environmental cues, and increase physiological readiness, thereby priming the organism for an adaptive fight-or-flight response if required [59,60]. This is why a dark places do not feel unpleasant only because of their appearance, but because vision does not provide enough reliable information for a safe evaluation of the space. When faces, passages, and distant objects are difficult to recognize, alertness increases and attention is more easily directed toward possible threats [6]. Spatial organization also influences emotional experience. Open, clear, and well-lit spaces are usually perceived as safer because they support orientation and prediction, while closed, crowded, or visually unclear spaces can more easily create tension [6,58]. Vision fulfills a critical adaptive role by facilitating spatial awareness and the real-time interpretation of environmental events. When this is compromised, the emotional system recalibrates toward a state of caution. Overall, these findings demonstrate that visual perception is closely linked to emotional processing, as elements such as color, light, and space influence how scenes are emotionally experienced and interpreted. The perceptual effects of visual atmosphere may arise from the modulation of early visual processing mechanisms, including those involved in contrast sensitivity and neural gain. Collectively, these mechanisms suggest that visual atmosphere extends beyond abstract cognitive appraisal by engaging a continuous neurobiological feedback loop. Through this process, retinal signals modulate subcortical and autonomic activity, shaping mood through ongoing interactions between physiological regulation and perceptual experience.

## 6. Uncertainty and Ambiguity

Uncertainty and ambiguity are important parts of the relationship between vision and emotion because unclear visual input leaves more room for emotional interpretation. When visual information is ambiguous, its interpretation can be shaped by threat-related biases, especially in individuals with higher anxiety or stronger negative interpretation patterns [61,62]. When a visual scene is unclear, the brain has to “fill in the gaps”, and this process is not always neutral, but can be influenced by a person’s emotional state and their tendency to interpret unclear stimuli as negative or threatening [63]. Under conditions of perceptual ambiguity, emotional factors may play a stronger role in shaping what we perceive, often biasing unclear stimuli toward possible threat. Research showed that a threatening context can alter the processing of emotional facial expressions [64]. Participants were slower and less accurate when they judged facial expressions under threat compared to safety, and happy or neutral faces were more often misclassified as fearful [64]. This suggests that threat can apply a negative filter to neutral and positive visual information, helping explain why the same facial expression may be interpreted differently when a person is already in a state of vigilance. Ambiguity is also important at the neural level. The amygdala rapidly encodes the valence of ambiguous affective stimuli, with these early neural representations showing significant variation according to individual anxiety symptoms [65]. Uncertainty regarding potential threats can bias perceptual decision-making, as while uncertain threat cues may enhance the detection of relevant information, elevated anxiety levels can impair the ability to accurately distinguish between threatening and neutral stimuli [66]. This suggests that emotional states can sometimes help perception by preparing the brain for danger, but they can also distort perception when anxiety pushes interpretation too strongly toward threat. This helps explain why unclear shadows, unfamiliar faces, or poorly visible movements can feel threatening before there is clear evidence of danger. In these situations, vision and emotion work together: vision provides incomplete information, while emotion helps the brain assign meaning to it. However, when information is unclear, this process often moves in a more cautious and negative direction, favoring a possible threat-related interpretation over a neutral one. In summary, perceptual ambiguity serves as a primary nexus where affective states modulate sensory interpretation. By filling gaps in incomplete visual information with threat-related priors, the brain demonstrates how anxiety and vigilance fundamentally bias the construction of reality, which further illustrates the bidirectional nature of the vision-emotion relationship.

## 7. Visual Stimuli and Emotional Memory

Visual stimuli are also closely connected with emotional memory. Images often leave a stronger impression than words because they are more direct, and easier to mentally recreate. When a visual experience is emotionally important, it usually attracts more attention, which can make the memory stronger and more vivid later. Research on emotional episodic memory shows that emotional images can improve memory for certain details of an experience, especially when the image has a strong personal or affective meaning [67]. Research indicates that emotions can enhance visuospatial memory when neutral and emotional stimuli compete for access to working memory, suggesting that emotional content is prioritized when visual information is limited or in competition [68]. This is why some visual memories remain very clear for a long time, such as frightening scenes, traumatic experiences, but also positive memories connected with important people, places, or events. Positive emotions can also strengthen associative memory, meaning that people may remember not only the image itself, but also the context and connections around it [69]. Attention also plays an important role in this process. Voluntarily directing visual attention toward an object can increase the perceived intensity of emotional reactions to that object [70]. In other words, what we visually focus on can become emotionally stronger. In the case of trauma, visual memory can become especially intense because emotional images may return as intrusive mental pictures rather than as ordinary verbal memories [71]. This is supported by research showing that threat conditioning can create intrusive memories that persist over time [72]. Research found that stronger stress responses before viewing traumatic material were associated with more frequent, vivid, and distressing intrusive memories later [73]. This shows that vision and emotion are strongly connected in memory: what we see can become emotionally meaningful, and that emotional meaning can make the visual memory more lasting, detailed, and personally important. Interplay between visual input and emotional memory illustrates how affective significance acts as a catalyst for memory consolidation. The system ensures that personally relevant experiences remain vivid through attentional and neurobiological reinforcement of emotionally salient images, which reinforces the central thesis that affective states function as fundamental architects of perceptual and memorial representation.

## 8. The Feedback Loop: Dynamic Interaction Between Vision and Emotion

Considering the described ways in which emotional states modulate visual processing, as well as the ways in which visual stimuli influence emotional states, the question arises as to how these mechanisms interact. Visual perception and emotions are functionally and neurally integrated within distributed networks in which, depending on the context, the same brain regions may participate in multiple functional processes [74]. Accordingly, this organization enables a continuous bidirectional interaction between perceptual and affective processes, whereby emotional states modulate visual processing through top–down mechanisms, while visually relevant stimuli shape affective evaluations and decisions [47].

The theory of constructed emotion proposes that emotions are predictive constructions generated through the integration of interoceptive signals, situational context, and conceptual knowledge derived from prior experience [75]. Affect, therefore, does not emerge from the activation of discrete and predefined emotional systems, but from inferential processes through which internal bodily states and contextual information are categorized and interpreted. In this sense, visual stimuli do not function as direct triggers of specific emotions. They act as input signals integrated into a broader predictive and inferential system that contributes to the construction of affective meaning. Consistent with this perspective, the interaction between vision and emotion is most apparent during the perceptual evaluation of emotionally salient visual content, where a combination of top–down attentional processes and bottom-up stimulus properties shapes interpretation and influences the speed of decision-making [76]. Modern analytical frameworks have further validated the predictability of these integrated systems. By utilizing linguistic and semantic parsing, current machine learning models can estimate underlying neuro-psycho-physiological affect with high precision, underscoring the deep structural link between perceptual throughput and emotional state [77]. This account of affective processing aligns with predictive coding and active inference theories, according to which the brain continuously minimizes prediction error by updating internal models on the basis of incoming sensory and interoceptive information [78]. While the theory of constructed emotion and the predictive processing frameworks utilized here offer a robust account of affective-perceptual integration, it is important to acknowledge that these represent only one lens within a diverse theoretical landscape. Alternative frameworks, such as Appraisal theory [79], emphasize the role of cognitive, stimulus-driven evaluations in shaping emotional response, while Embodied cognition theories highlight the foundational role of somatic and sensorimotor feedback in grounding emotional experience [80]. These perspectives provide complementary insights. For instance, whereas predictive processing focuses on the computational minimization of uncertainty, appraisal and embodied models offer crucial focus on the functional-cognitive and physiological precursors of affect. A comprehensive understanding of the vision-emotion loop likely requires a synthesis that integrates these computational, cognitive, and bodily perspectives.

Taken together, these perspectives suggest that emotions and visual perception are linked through a continuous directional interaction, in which perceptual input contributes to the construction of affective meaning, while affective states shape the interpretation of visual information. Rather than functioning as independent systems, vision and emotion appear to operate as mutually constraining components of adaptive cognition.

## 9. Continuous Bidirectional Influence: Predictive Coding and Active Inference Models

The interaction between affective states and visual perception can also be understood through predictive processing theories, which propose that the brain continuously relies on internal models of the world to anticipate incoming sensory information [81]. More specifically, predictive coding describes a hierarchical organization in which higher cortical regions generate predictions that are transmitted to lower sensory areas, while lower levels compare these expectations with actual sensory input and compute discrepancies between them, known as prediction errors [81]. Rather than passively receiving information from the environment, the brain continuously compares incoming sensory signals with internally generated predictions, updating them whenever mismatches occur [78,81,82].

Emotions and affective states may therefore be viewed as integral components of the same predictive system responsible for generating and updating perceptual models, rather than as processes occurring only after perception has taken place. Contemporary predictive processing accounts consequently describe emotions not as isolated affective reactions, but as functional components of a generative mechanism that continuously shapes perception itself [74,83]. This complex physiological relationship presents notable computational utility. Emerging biomathematical and machine learning frameworks increasingly utilize linguistic and semantic parsing to estimate underlying neuro-psycho-physiological affect, underscoring the predictability of these integrated systems [77].

Consistent with the theory of constructed emotion, emotional states arise from predictive constructions integrating interoceptive and exteroceptive signals within hierarchical brain systems [75]. Affective and interoceptive processes may therefore contribute to the modulation of predictive expectations regarding incoming visual signals, particularly under conditions of uncertainty or perceptual ambiguity [82,84,85]. For example, anxious affective states may increase expectations of threat, resulting in biased interpretations of neutral stimuli. Predictive processing models interpret such effects as adaptive inferential processes under uncertainty, in which affective and interoceptive states shape perceptual hypotheses [75,82,84]. For example, an individual experiencing increased anxiety may interpret an ambiguous facial expression as threatening because threat-related expectations have a stronger influence on perceptual inference. Conversely, positive affective states may facilitate more benign interpretations of uncertain visual information, illustrating how emotional states shape perceptual hypotheses under conditions of ambiguity.

A central mechanism in this process is precision weighting, referring to the estimation of the reliability of prediction error signals and determining the extent to which sensory evidence, relative to prior expectations, influences perceptual inference, belief updating, and learning [86]. In simple terms, precision weighting determines whether perception is guided more strongly by what is currently being seen or by what is expected to be seen. Emotional states may modulate this process by altering the relative precision assigned to sensory information and internally generated predictions [84,87]. Emotions can further be understood as forms of allostatic anticipation, in which interoceptive and affective signals dynamically regulate the integration of internally generated predictions and external sensory input within hierarchical predictive systems [83,84,87]. In this context, anxiety has been associated with heightened precision of threat-related predictions and increased sensitivity to uncertainty, whereas depressive states are often characterized by rigid negative priors and reduced flexibility in updating beliefs [88,89,90,91].

**Figure 1:**
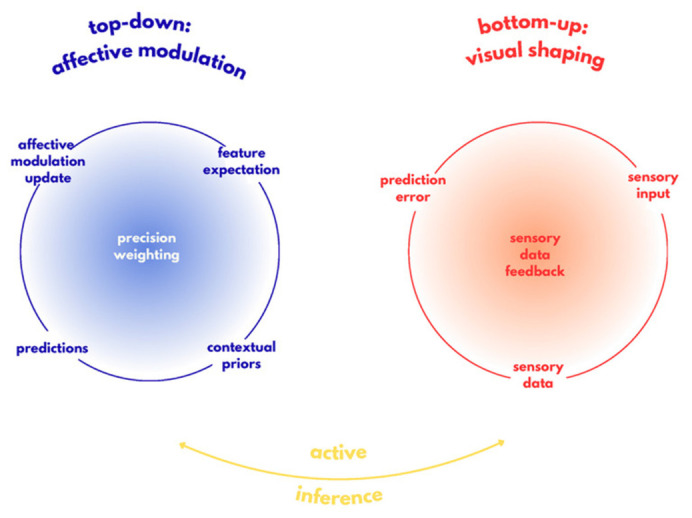
Integration of Top-Down Affective Modulation and Bottom-Up Sensory Processing within a Bidirectional Predictive Framework.

These ideas extend into active inference models, according to which organisms minimize prediction error not only by updating internal perceptual models, but also by acting upon the environment in ways that modify sensory input [92]. Perception and action therefore form a unified closed-loop system through which organisms actively shape their environment in order to reduce uncertainty and test perceptual hypotheses regarding the causes of sensory input [93,94]. On the behavioral level, eye movements, attentional orienting, and approach or avoidance behaviors function as mechanisms for gathering information, optimizing perception, and minimizing prediction error [95,96]. As illustrated in Figure 1, the described mechanisms are integrated within the active inference framework, in which perception emerges from a continuous interaction between top–down affective modulation and bottom-up sensory processing through the ongoing updating of predictions, contextual priors, precision weighting, sensory input and prediction errors to minimize uncertainty.

This principle also applies to behavior under uncertainty, where protective behaviors may be interpreted as strategies aimed at reducing discrepancies between expected and actual bodily states, thereby maintaining internal stability and predictability [97]. Similar principles may extend to communication processes, which can be viewed as exchanges of predictions intended to reduce uncertainty and coordinate behavior, with emotions functioning as tools for alignment with the internal models of others [98]. Taken together, emotions influence not only the interpretation of sensory stimuli, but also the selection and weighting of sensory information through modulation of inferential precision signals [84,93,99]. In summary, predictive coding and active inference offer a coherent framework for understanding how perception, emotion and action emerge through continuous bidirectional interaction. At the same time, these models remain evolving theoretical frameworks whose explanatory power may be enhanced through integration with complementary perspectives.

## 10. Temporal Dynamics: Millisecond-Scale Neural Responses vs. Long-Term Mood–Vision Cycles

In addition to the multilevel and dynamic mechanisms underlying the interaction between emotions and visual perception, it is important to consider the temporal scales across which these interactions unfold, ranging from rapid neural responses to slower cumulative changes in affective and perceptual patterns [100,101]. Such organization reflects the integration of perceptual and emotional processes within distributed and dynamically interconnected neural networks [102].

At the fastest levels of processing, emotionally salient visual stimuli may undergo rapid affective evaluation through subcortical pathways, enabling the prioritization of biologically relevant information even before complete conscious appraisal [47]. Empirical findings and review studies further demonstrate that emotional content modulates early stages of visual processing within the visual cortex, suggesting that affective significance influences perceptual elaboration from its earliest stages. Rapid emotional responses therefore occur in continuous interaction with slower distributed neural processing, while bottom-up sensory input is simultaneously shaped by top–down influences such as attention, expectations, and current affective states [8].

Such dynamics support processes unfolding across seconds and minutes, including selective attention and contextual reinterpretation of visual stimuli, mediated through ongoing reorganization of distributed neural networks [100,102]. Prolonged exposure to stress or persistent negative affective states may additionally lead to neurobiological changes within prefrontal and limbic networks, reducing top–down attentional control and contributing to increased attentional bias toward threat. Over time, repeated activation of these mechanisms may stabilize into enduring perceptual patterns [103,104,105].

At longer temporal scales encompassing days, months, or years, moods and clinical conditions exhibit relative stability and may shape enduring patterns of emotional reactivity and cognitive processing [106]. Depression, for example, may contribute to stable cognitive and affective patterns that influence the interpretation of perceptual information through persistent predictive beliefs and reduced flexibility in updating them [90], whereas anxiety is associated with chronically heightened sensitivity to threat and interpretative biases [89].

Contemporary affective neuroscience describes these temporal levels as part of a continuous hierarchical system in which rapid sensory signals and slower affective models continuously interact and shape one another [100,101]. Emotional states are therefore not merely consequences of perceptual processing, but active contributors to how visual information is interpreted over time [101]. Taken together, these findings suggest that the vision-emotion relationship unfolds across multiple temporal scales, from millisecond-level neural responses to enduring mood-related perceptual biases, reinforcing the concept of a dynamic bidirectional interaction between affective and perceptual systems.

Over time, the continued interplay between emotional and perceptual systems may be reflected in measurable changes in neural and ocular functioning, offering a possible link between basic psychological mechanisms and clinically relevant applications.

## 11. Retinal and Ocular Changes in Neurodegenerative and Mood Disorders

The previously described visual and neural processes, along with their impact on emotions and perception, are of paramount importance in the study of psychopathology. These processes constitute a significant aspect of daily life, and any disruptions to them can lead to substantial suffering in cognitive and emotional functioning. Indeed, clinical investigations specifically tracking this intersection confirm that progressive ocular pathologies directly correlate with a measurable reduction in life quality and an elevated risk profile for co-occurring mood or mental health difficulties [107]. As will be further examined, anatomical and physiological alterations in the eyes and neural pathways can result in significant challenges in an individual’s life.

A rapidly emerging area of research is the connection between the eye and the brain, changes that can occur in their anatomy, and the consequences of those changes relating to mood and neurodegenerative diseases. Studies report alterations of vision, structure, and function in the retina and the brain, as well as the presence of different hallmarks of disease. Important implications can be found in such studies: the interaction between vision and the brain and the possibility of using a wide range of eye-tracking and retinal imaging methods for the detection of mental health or neurodegenerative disorders [108]. A new growing field made possible by advancements in retinal imaging in combination with large retinal data sets is called oculomics [109,110]. The most important aspect of oculomics is the possibility of examining indicators of mental health, brain health, and neurological disorders on a molecular, cellular, and structural level, all through the eye. It is an essential pathway where the brain translates outside stimuli into an image.

The eye is considered a part of the central nervous system due to the direct connection to the brain called the optic nerve or the second cranial nerve. The posterior part of the eye is called the retina, comprising ten layers and several different cell types with important roles in light detection and visual input. When light enters the eye, sensory neurons or photoreceptors convert it into a signal by changing the membrane potential and the transmission of neurotransmitters. Different pathological alterations to the retina can change the number of photoreceptors, more specifically cones and rods. Furthermore, ganglion cells are the last step in visual processing by transmitting stimuli to the optic nerve and have been important in diagnosing glaucoma, using optical coherence tomography (OCT) [111]. One of the parts of the retina is the retinal pigment epithelium (RPE), a single layer of cells and a fundamental part of the retina with essential visual functions [112]. The RPE also has an important role in the development of retinal diseases, as well as being a critical element of the blood-retina barrier and in the maintenance of retinal homeostasis. It allows for the constant exchange of nutrients, signaling molecules, and metabolic products. The ion composition in the sub-retinal space is maintained by the RPE and can be disrupted by some diseases. For example, a central characteristic of Alzheimer’s disease (AD) is retinal texture that can be observed by optical coherence tomography [111].

As previously stated, changes in the anatomical structure of the eye are associated with a number of mood and neurodegenerative disorders, such as Alzheimer’s disease and major depressive disorder (MDD). Alzheimer’s disease is the most common form of dementia, taking up approximately 60% to 80% of all cases [113]. One of the Alzheimer’s disease hallmarks is the existence of amyloid-beta in the retina and tau intracellular tau tangles in neurons, which are used for post-mortem diagnosis [114]. The plaque is formed by producing an unfolding of amyloid peptide fragments called amyloid-β protein (1–40) and amyloid-β protein (1–42). They are accumulated in the retina with age, causing neurodegenerative changes in the retinal ganglion cell layer [111]. By examining the retinal tissue of patients with Alzheimer’s disease in comparison to a control group, one study identified a specific regional distribution of amyloid-beta within the retinal ganglion cell layer, finding higher concentrations in the mid-periphery and noting the most significant differences between Alzheimer’s and control retinas in the superior and temporal quadrants [114]. The retinal and cortical deposition of amyloid-beta proteins can be better understood by animal models. Parallel changes were found in both amyloid-beta and neurotransmitters, as well as synaptic remodeling and microglial activation [115]. Furthermore, another change in functioning of the retina in AD patients can be found in the pupil light reflex, the response of the pupil due to the presence or absence of light. Most studies of the pupillary response present the cholinergic deficit in patients with Alzheimer’s disease compared to the control group, showing alterations in pupil diameter, amplitude, pupillary response velocity and acceleration, and constriction latency. However, research into pupillary responses is often hindered by high variability and limited sample sizes [113]. Another potential method of AD diagnosis has been tracking eye movements (EM). Although they are not considered primary signs of Alzheimer’s disease, various abnormalities have been identified in AD patients, differing according to the stage of the disease. Functions affected are mostly gaze and visual fixation, which result in limited shifting of focus and field of view. Cognition and gaze patterns are shown to be correlated [113]. Furthermore, increased latency, reduced acceleration, and decreased velocity were found in patients with dementia, as well as a strong correlation between dementia severity and visual tracking abnormalities in patients with AD.

Beyond the perceptual and attentional abnormalities discussed earlier, increasing evidence suggests that major depressive disorder exerts a multi-level influence on the visual system, extending from functional electrophysiological disruptions to measurable structural and vascular alterations in the retina. At the most fundamental level, studies have shown that patients experiencing visual impairment without visual acuity loss have double the risk of depression, either due to brain-vision axis changes or other psychological factors, suggesting a bidirectional relationship between mood and visual processing. This functional disruption is further supported by electroretinogram (ERG) findings, which assess retinal function by measuring electrical activity in response to light exposure, revealing differences primarily in rods and cones [116]. Using PERG (pattern electroretinogram), MDD patients showed lower retinal contrast gain compared to healthy individuals, with studies demonstrating a strong correlation between depression severity and retinal contrast gain—pointing toward a potential prognostic biomarker at the level of early visual signal processing [116]. However, this evidence must be interpreted cautiously: the key study included only 40 patients, limiting generalizability, and given its pilot nature, comparable data on patients with schizoaffective disorder or schizophrenia are lacking, meaning the specificity of the biomarker cannot yet be determined [117].

Structural imaging studies using OCT further support the notion that MDD affects the retina at multiple levels, though with less consistency. While electrophysiological markers often reflect rapid, functional shifts in neural activity [118], structural integrity measured via OCT may represent the cumulative, downstream effects of these prolonged neuro-metabolic stressors [119,120]. These modalities likely operate on different temporal scales, with functional dysregulation potentially preceding detectable structural thinning in the progression of depressive pathology. Mixed findings have been reported: while some studies identified significantly reduced ganglion cell layer, inner plexiform layer, and global and temporal retinal nerve fiber layer thickness in MDD patients compared to healthy controls, the majority of evidence suggests that OCT measures do not differ significantly between patients with major depression and healthy controls overall, with more substantial differences emerging within MDD subgroups. Where differences have been observed, negative correlations between the ganglion cell layer and inner plexiform layer thickness and the duration and severity of MDD suggest that structural retinal changes may accumulate with disease burden, while positive correlations between global retinal nerve fiber layer thickness and depression severity further indicate that the relationship between MDD and retinal morphology is nuanced and likely state-dependent [116]. Extending beyond neural layers, another study found differences in retinal vascular density, choroidal thickness, and visual acuity in MDD patients compared to healthy controls using OCT [121], situating MDD’s retinal impact within a broader vascular context. This is reinforced by the elevated rates of glaucoma, dry eye syndrome, and retinal nerve fiber layer thinning observed in MDD, likely mediated by pharmacological factors, vascular dysregulation, and systemic inflammation [122]. OCTA studies further corroborate this vascular dimension, finding reduced vessel density in MDD and pointing toward neurovascular impairment possibly driven by inflammatory processes causing microvascular blockage and decreased retinal blood flow [121]. Taken together, these findings spanning electrophysiological signal processing, structural layer integrity, and retinal vasculature converge to suggest that MDD does not affect the visual system through a single mechanism but rather disrupts it across multiple levels. Nonetheless, given the small sample sizes, as in one functional study limited to 31 participants [120], and the inconsistency of findings across methods, these results should be regarded as preliminary indications rather than established markers of disease. This multi-level retinal disruption reflects the systemic nature of major depressive disorder. Given the uninterrupted anatomical lineage between the retina and the brain, depression should be viewed as a multimodal disorder characterized by interconnected metabolic and proteomic dysfunctions across the entire brain-body axis [123].

While amyloid-beta deposition and retinal nerve fiber layer (RNFL) thinning are key clinical indicators, they are downstream manifestations of earlier molecular dysfunction. Recent evidence points to dysregulated pre-mRNA alternative splicing as a fundamental antecedent. Such transcriptomic alterations can lead to aberrant protein isoforms that compromise synaptic integrity and cellular homeostasis well before macroscopic atrophy is detectable [124]. By linking these splicing dynamics to early retinal dysfunction, we establish a clearer mechanistic bridge between molecular neurodegeneration and the observable visual aberrations that characterize these clinical states.

While oculomics and retinal imaging hold considerable promise as non-invasive windows into brain and mental health, it is important to recognize that the field remains in its early stages and that clinical translation faces substantial challenges. Much of the current evidence is derived from small, heterogeneous samples, as noted in several studies cited above, which limits statistical power and generalizability. Beyond sample size concerns, a central methodological obstacle is specificity: retinal changes such as thinning of the ganglion cell layer or reduced vessel density are not unique to any single disorder, appearing across Alzheimer’s disease, major depressive disorder, glaucoma, and other conditions, making it difficult to establish disease-specific biomarkers [120]. Technological variability between imaging devices and protocols further complicates cross-study comparisons and standardization. Additionally, most findings to date are correlational and cross-sectional, meaning causal directionality between retinal changes and neuropsychiatric pathology remains unclear. Longitudinal studies with larger, more diverse cohorts and standardized imaging protocols are therefore necessary before retinal biomarkers can be considered reliable diagnostic or prognostic tools. In sum, although oculomics represents an exciting and rapidly developing frontier, claims regarding its diagnostic utility should be interpreted with caution until the translational gap between exploratory research and clinical application is more rigorously addressed.

## 12. Future Directions

Future research should pursue several converging lines of inquiry to deepen understanding of the bidirectional relationship between emotion and visual perception.

First, further investigation of the underlying neurobiological mechanisms is needed. At the neural level, combining the temporal resolution of EEG with the spatial precision of fMRI will allow researchers to more precisely map how rapid subcortical responses mediated by the superior pathway interact with slower top–down modulatory influences from prefrontal and limbic networks, clarifying which aspects of emotional modulation are truly automatic versus attention-dependent [8,47,125]. Future studies should also examine the long-term neural consequences of chronic stress on prefrontal-limbic connectivity and perceptual flexibility, as these alterations may further contribute to maladaptive emotion-perception interactions [105].

Second, future research should continue refining theoretical and computational models of emotion-perception interactions. Predictive processing and active inference frameworks offer a promising foundation for formalizing how affective states modulate precision weighting and perceptual updating. These computational models should be empirically validated across both healthy and clinical populations, including those with anxiety, depression, and PTSD, where maladaptive perceptual biases are particularly pronounced [75,84,90,91].

Third, greater emphasis should be placed on the clinical translation of these findings. Retinal imaging and eye-tracking hold potential as non-invasive biomarkers of affective disorders and neurodegenerative disorders. In particular, advance in optical coherence tomography (OCT) have enabled high-resolution visualization of retinal structures, allowing more sensitive detection of structural changes, especially within the ganglion cell layer (GCL) [116]. Such biomarkers may facilitate earlier diagnosis of disorders including major depressive disorder and Alzheimer’s disease, improve treatment planning and complement existing behavioral and neuroimaging criteria in future diagnostic models [122]. Finally, future research should pay greater attention to sources of individual variability and ecological validity. Factors such as age, cultural background and neurodiversity may influence threat detection, attentional allocation and the interpretation of emotionally ambiguous visual information, contributing to differences in perceptual biases across populations and contexts. Future studies should also explore immersive approaches, such as virtual reality to investigate emotion-perception interactions under more naturalistic conditions. To transition from preliminary evidence to robust clinical application, future research should prioritize the integration of ocular metrics with large-scale, open-source epidemiological and neuroimaging datasets. Such multi-modal cross-validation is essential to establish the reliability and transdiagnostic specificity of these biomarkers across diverse, population-level cohorts. Collectively, these directions call for a more integrated and clinically oriented science of emotion and perception—one that treats the visual system not as a passive recorder of external reality, but as an active, affectively shaped system whose functioning is deeply embedded in the emotional life of the organism [75,102].

## 13. Conclusions

The transition from viewing vision as a passive, objective process to an active, inferential system represents a major advancement in cognitive science. As synthesized across this review, the visual system does not merely map the objective coordinates of the physical world; it actually actively constructs an environmental landscape colored by internal emotional demands, evolutionary imperatives, and homeostatic needs. This continuous bidirectional loop relies on an intricate neural architecture where rapid, survival-salient subcortical appraisals interact seamlessly with slower, detailed cortical interpretations. When filtered through predictive processing models, emotions emerge not as secondary reactions to sensory data, but as foundational precision weights that continuously recalibrate perceptual hypotheses and modulate physical action within the environment.

Recognizing this deep functional unity between sensory and affective processing has profound clinical ramifications. Because the eye shares an uninterrupted anatomical and physiological lineage with the central nervous system, localized variations in visual processing, ranging from altered cortical contrast suppression to molecular and structural retinal degeneration, serve as early indicators of broader psychopathological and neurodegenerative distress. Oculomics is an emerging discipline that may allow for the development of scalable, non-invasive screening tools based on these findings. However, further studies are needed to validate the reliability and clinical applicability of these approaches before they can be incorporated into routine clinical practice. Moving forward, the primary challenge for the field lies in translating these complex biomathematical and ocular markers into validated screening protocols capable of catching mood disorders and neurodegenerative pathologies long before downstream behavioral or cognitive deficits fully crystallize. Ultimately, by viewing vision through the lens of feeling, cognitive neuroscience moves closer to an integrated, holistic model of human experience, blurring the traditional boundaries between perception, emotion, and biological reality.

## Data Availability

No new data were created or analyzed in this study.
